# Identification of new genes in *Sinorhizobium meliloti *using the Genome Sequencer FLX system

**DOI:** 10.1186/1471-2180-8-72

**Published:** 2008-05-02

**Authors:** Chunhong Mao, Clive Evans, Roderick V Jensen, Bruno WS Sobral

**Affiliations:** 1 Virginia Bioinformatics Institute, Virginia Polytechnic Institute and State University, Blacksburg, VA 24061, USA

## Abstract

**Background:**

*Sinorhizobium meliloti *is an agriculturally important model symbiont. There is an ongoing need to update and improve its genome annotation. In this study, we used a high-throughput pyrosequencing approach to sequence the transcriptome of *S. meliloti*, and search for new bacterial genes missed in the previous genome annotation. This is the first report of sequencing a bacterial transcriptome using the pyrosequencing technology.

**Results:**

Our pilot sequencing run generated 19,005 reads with an average length of 136 nucleotides per read. From these data, we identified 20 new genes. These new gene transcripts were confirmed by RT-PCR and their possible functions were analyzed.

**Conclusion:**

Our results indicate that high-throughput sequence analysis of bacterial transcriptomes is feasible and next-generation sequencing technologies will greatly facilitate the discovery of new genes and improve genome annotation.

## Background

*Sinorhizobium meliloti *is a micro-symbiont associated with legume plants. This soil bacterium inhabits nodules on the roots of host legume plants, where it reduces atmospheric nitrogen to organic nitrogenous compounds that can be utilized by its hosts. Because of its agricultural and ecological importance, *S. meliloti *has been extensively studied as a model symbiont. The *S. meliloti *1021 genome sequence and the initial annotation of the genome were completed in 2001 [1-4]. The *S.meliloti *genome comprises three replicons, the 3.65 Mb chromosome, the 1.35 Mb megaplasmid pSymA, and the1.68 Mb megaplasmid pSymB [4]. According to RefSeq [5], the *S. meliloti *1021 genome has 6205 predicted protein-encoding genes. Among these, more than one-third were annotated as "hypothetical" or "unknown". Many research papers have been published on *S. meliloti *since its genome sequence was completed. Also, more genomes of closely related species such as *Brucella *spp., *Rhodopseudomonas palustris*, and *S. medicae *WSM419 have been sequenced. Comparative genomics including newly sequenced genomes provides new information about the genome of *S. meliloti*. There is an ongoing need to update and improve its genome annotation. So far, there are no systematic efforts of direct sequencing of its entire transcriptome. Microarray data are available, but most microarray designs are based on annotated genes [6,7]. High-density whole-genome tiling arrays are not yet available.

The goal of this study was to develop a high-throughput experimental approach to search for new genes of *S. meliloti *missed in the previous genome annotation [1-4]. We used pyrosequencing [8] to sequence the transcriptome of *S.meliloti*. The GS FLX system from Roche and 454 Life Sciences can generate more than 100 million bases per sequencing run with an average yield of greater than 400,000 reads of average length of 250 bases. This platform provides a broad range of applications including whole genome sequencing [9-11], transcriptome and gene regulation studies [12-15], metagenomics analysis [16] and amplicon sequencing [17,18]. Although pyrosequencing has been used to sequence microbial genomes, relatively few applications of transcriptome analysis have been reported. Here, we present the first report of sequencing a bacterial transcriptome using the GS FLX platform as an experimental approach for gene discovery.

## Results

### Gene prediction

**Figure 1:**
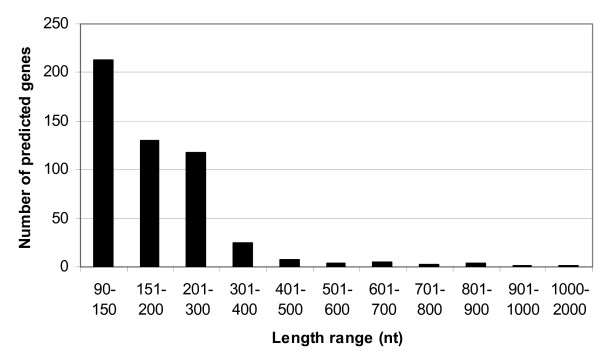
Length distribution of predicted gene candidates.

We used an automated gene annotation pipeline provided by PATRIC [19] to predict genes in *S. meliloti*. This pipeline uses a combination of gene prediction programs, Glimmer [20], GeneMark [21,22], TICO [23] and RBSfinder [24] to predict genes and compares with genes in RefSeq. A total of 512 new protein-coding genes (with length >90 nt) in the intergenic regions of the genome were predicted through this automated pipeline (Additional file 1). The number of predicted genes in different length ranges is shown in Figure 1. Most of the predicted new genes are relatively small (length <400 nt). The average length is about 200 nt. These genes were BLASTed against the NCBI non-redundant (NR) protein database [25,26]. The result showed that 159 candidates had BLAST hits in the NR database with E-values less than 0.01, whereas the remaining 353 of the candidates had no significant hits. Small gene size and lack of BLAST hits may be the reasons that the predicted new gene candidates were missed in the original genome annotation process.

### Sequence analysis

**Figure 2:**
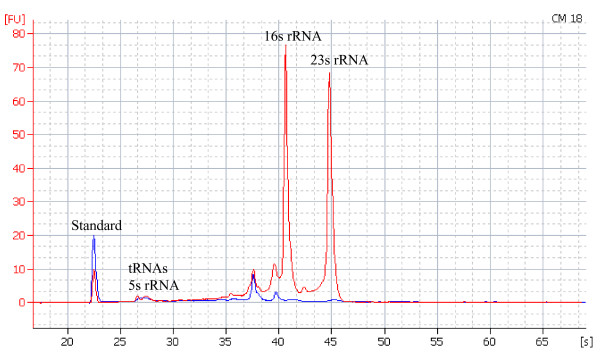
**Removal of 16s and 23s rRNAs using MICROB *Express*™ kit from Ambion**. Total RNA samples before (in red) and after (in blue) rRNA depletion were analyzed on the Agilent 2100 Bioanalyzer.

Total RNAs were extracted from *S. meliloti *1021 cells grown to mid-exponential phase in the TY medium and treated with DNase I to remove genomic DNA (Methods). The 16s and 23s rRNAs were depleted and the RNA samples were then amplified to produce cDNA fragments of average length about 150 nt (Methods). With two test cDNA samples loaded on to 4 lanes per sample of a 16 lane sequencing plate, the titration run generated a total of 19,005 high quality reads with average length of 136 nt (Table 1). Although our rRNA removal step indicated that more than 90% rRNAs were depleted as judged by Agilent 2100 Bioanalyzer, approximately 90% of the reads still aligned to the rRNA operons (Figure 2). This may be due to relative low mRNA population in the *S. meliloti *cells. Out of 17092 reads aligned to the rRNA operons, 3 reads matched 5s rRNA, 2860 reads matched 16s rRNA, 13983 reads matched 23s rRNA, and the remaining 246 reads aligned to the integenic regions between the rRNA genes in the rRNA operons. For the 1854 non-rRNA sequences, 1774 matched to 737 of the 6271 RefSeq genes (proteins and RNAs) and 59 matched to 32 of 512 new protein-coding genes predicted through our gene prediction pipeline. The remaining 21 sequences mainly matched sequences either immediately before or after a coding region, presumably 5' UTR or 3' UTR.

**Table 1:** GS FLX sequencing results

	Sample 1	Sample 2	Total
# Sequence reads	8694	10311	19005
Average sequence length	139	133	136
# Sequences aligned to genes	1165	689	1854
# Sequences in rRNA operons	7513	9579	17092
# Sequences not aligned to the genome (e<0.01)	16	43	59

### Validation of new genes using RT-PCR

**Figure 3:**
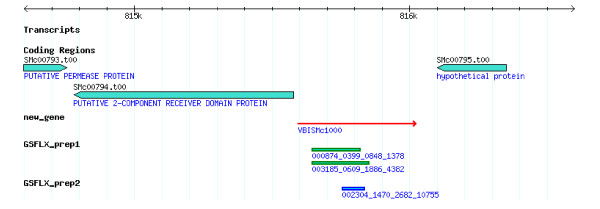
**Genome view of a new gene (in red)**. GLX sequences (in green from prep 1 and in blue from prep 2) are aligned to VBISMc1000.

**Figure 4:**
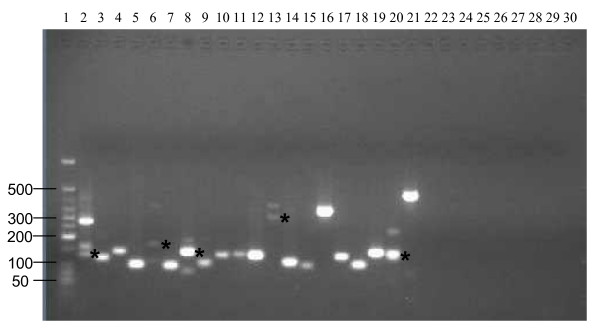
**RT-PCR of 20 gene candidates**. Lane 1: low molecular weight DNA ladder from New England Biolabs. Size range: 25 bp to 766 bp. Lane 2-21: 20 gene candidates, VBISMa0080, VBISMa0492, VBISMa1337, VBISMb0078, VBISMb0839, VBISMc0095, VBISMc0802, VBISMc1000, VBISMc1221, VBISMc1492, VBISMc1793, VBISMc2171, VBISMc2174, VBISMc2596, VBISMc2940, VBISMc2955, VBISMc3188, VBISMc3282, VBISMc4046 and VBISMc4289, respectively. For majority RT-PCR reactions, each produced one corresponding PCR product (lane 3-5, 7, 9-12, 14-19 and 21). These PCR products were directly sequenced and their sequences matched to the corresponding gene candidates. Multiple PCR products were found in lane 2, 6, 8, 13 and 20. The bands with the correct PCR product sizes are labeled with *. These PCR products were used to do a second round of PCR to produce enough DNA for sequencing. The sequencing results confirmed that they matched to the corresponding gene candidates. The most abundant PCR product in lane 2 was sequenced and determined to be a part of 23s rRNA sequence. Lane 22-29: negative controls using the RNA sample that was not reverse transcribed and primer pairs of new genes to show no genomic DNA contamination. In each lane of 22 to 29, combined primer pairs of two or three genes were used. Lane 30: no template control. Primer pairs of cm0012a, cm012b and cm016a, cm016b were used.

Twenty new gene candidates with multiple GS FLX sequence hits or long sequence hits (>80 nt) were chosen for further verification using RT-PCR analysis of the original total RNA samples. These new gene candidates were predicted by PATRIC pipeline as protein-coding genes. Figure 3 shows an example of a new gene candidate (VBISMc1000) in the *Sinorhizobium *Genome Browser, which was built using the GBrowse software [27]. All 20 genes were detected in the RT-PCR experiment and the PCR products were sequenced and confirmed (Figure 4). The negative controls indicated that there was no genomic DNA contamination in the RNA samples tested.

**Figure 5:**
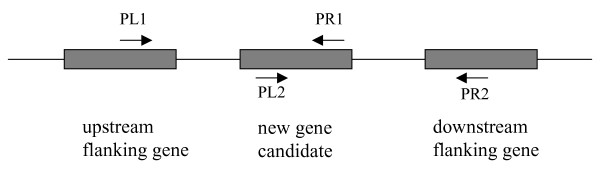
**Primer design for testing co-transcription using RT-PCR**. PL1 and PR1 are primer set for testing transcript from upstream flanking gene to new gene and PL2 and PR2 are primer set for testing transcript from new gene to downstream flanking gene.

To test whether the new genes are co-transcribed with their upstream or downstream flanking genes (if any), a set of primer pairs were designed (Figure 5, Additional file 2) to detect RT-PCR product of the transcripts with the flanking genes. The results are summarized in Table 2. Columns "co-transcribed with upstream gene" and "co-transcribed with downstream gene" indicated whether the RT-PCR products of the transcripts, which span from the upstream flanking gene to the new gene or from the new gene to downstream flanking gene, were detected. Ten of the predicted genes were not detected to be co-transcribed with either upstream or downstream flanking genes (Table 2).

**Table 2:** Summary of new genes

**Gene**	Replicon*	**Predicted start**	**Predicted end**	**Strand**	**Length**	**Co-transcribed with upstream gene**	**Co-transcribed with downstream gene**	**Target description for predicted genes**	**E-value**	**Percent similarity**
VBISMa0080	A	65258	65494	-	237	SMa0121	-	-	-	-
VBISMa0492	A	410418	410660	+	243	-	-	-	-	-
VBISMa1337	A	1191271	1191525	-	255	-	-	putative dioxygenase, slightly similar to catechol 1,2-dioxygenase protein [*S. meliloti *1021]	2e-4	25
VBISMb0078	B	66440	66619	+	180	-	SMb20056	hypothetical protein BMEII0534 [*B. melitensis *16M]	4e-3	30
VBISMb0839	B	679069	679800	+	732	-	-	two component transcriptional regulator, LuxR family [*S. medicae *WSM419]	2e-68	69
VBISMc0095	C	83906	84025	+	120	tRNA-Ala	23s rRNA	-	-	-
VBISMc0802	C	662922	663287	+	366	-	-	-	-	-
VBISMc1000	C	815598	816023	+	426	-	-	hypothetical protein Smed_0338 [*S. medicae *WSM419]	6e-20	36
VBISMc1221	C	997383	997565	+	183	ctaE	SMc00014	hypothetical protein Smed_0524 [*S. medicae *WSM419]	1e-15	63
VBISMc1492	C	1208848	1209090	-	243	-	-	-	-	-
VBISMc1793	C	1444596	1444832	-	237	-	rne	-	-	-
VBISMc2171	C	1725819	1726607	-	789	-	SMc01204	hypothetical protein Smed_1270 [*S. medicae *WSM419]	8e-124	89
VBISMc2174	C	1728081	1728221	-	141	SMc01200	SMc01202	-	-	-
VBISMc2596	C	2060940	2061143	+	204	-	csp4	-	-	-
VBISMc2940	C	2312866	2313225	-	360	-	-	conserved hypothetical signal peptide protein [*S. medicae *WSM419]	2e-54	89
VBISMc2955	C	2321074	2321289	+	216	-	-	-	-	-
VBISMc3188	C	2520862	2520993	-	132	-	-	-	-	-
VBISMc3282	C	2595016	2595537	+	522	-	SMc01535	Nodulation protein nolR	9e-50	59
VBISMc4046	C	3233965	3234153	+	189	SMc03108	-	hypothetical protein pRL110117 [*R. leguminosarum *bv. viciae 3841]	1e-5	56
VBISMc4289	C	3417257	3419215	-	1959	-	-	hypothetical protein Cvib_0070 [*P. vibrioformis *DSM 265]	4e-35	47

*: replicon A: pSymA; B: pSymB; C: Chromosome.

### Functional annotation of the new genes

The sequences of putative new genes were searched against the NR database from NCBI and SwissProt from EBI [28] using BLASTX [25] and Smith-Waterman programs ([29]; Table 2). Both programs produced very similar results: 10 of the 20 new genes had no significant hits, and the other 10 had either a full length or partial match with proteins in the NR database (Table 2). The genes with no significant hits were relatively short with lengths ranging from 120 to 366 nt.

Four predicted genes showed significant matches with genes with known or putative functions (Table 2). VBISMc2940 had 89% similarity to a conserved hypothetical signal peptide protein from *S. medicae *WSM419. VBISMb0839 had 69% similary to a two component transcriptional regulator, LuxR family from *S. medicae *WSM419. VBISMa1337 partially matched to a putative dioxygenase with 25% similarity. VBISMc3282 matched to nodulation protein NolR with 59% similarity. The NolR protein is a transcriptional regulator for common nodulation genes as well as the three nodD copies present in *S. meliloti*. Previous studies have shown that nolR gene in *S. meliloti *strain 1021 has a single insertion in the C-terminal coding sequence which abolishes the DNA-binding ability of the NolR protein [30,31]. Thus, Rm1021 has no NolR activity. NolR^- ^strains nodulate host plants less efficiently than NolR^+ ^strains. In the RefSeq database, VBISMc3282 was not previously annotated as a gene although it is a mutant form of the *nolR *gene. Here, we demonstrate and confirm that this mutant gene is expressed.

No neighboring genes were detected to be co-transcribed with VBISMc2940, VBISMb0839 or VBISMa1337, while VBISMc3282 was co-transcribed with the downstream gene SMc01535, a hypothetical protein. The other six genes with BLAST hits matched only hypothetical proteins (Table 2).

### Gene expression levels

Because the RNA amplification step was linear (Methods), we expect that the cDNA samples we prepared represent relative mRNA levels in the cell. Thus, for a full sequencing run, with high coverage, the number of sequences would be a good indication of gene expression levels. Due to the low coverage of our pilot experiment, we cannot yet estimate gene expression levels based on number of sequences for each gene. However, we expect that most of the genes that showed five or more matches to our transcriptome sequences should be highly expressed in the cell population (Additional file 3). The known genes with high sequence copy number are consistent with our knowledge about the high expression level of those genes under the same growth condition (our unpublished microarray data).

## Discussion

Our study demonstrated that there are many genes missed in the initial genome annotation and it is useful to have large-scale transcriptome analysis to reveal these genes and validate their status. Our results showed that sequencing bacterial transcriptomes using the GS FLX system is feasible and it helps to discover new genes and improve the genome annotation. A full GS FLX sequencing run can produce an average yield of more than 400,000 reads which is 20-fold greater than the yield from our titration run for this study. Even with 90% rRNA population in the sample, there will still be more than 40,000 reads that are non-rRNA transcripts. This provides an average 6X coverage of non-rRNA genes. Our pilot experiment with only 1854 reads already identified 20 new genes. With a full sequencing run, which produces more than 20-fold reads than the titration run, we expect to discover many more new gene transcripts that have been previously missed. However, a full sequencing run with 6X coverage of non-rRNA genes will still not be sufficient to discover all possible new genes expressed, especially for ones with low expression levels, and considering that conditions under which genes are expressed may not be known or studied by any particular set of experiments. According to the previous microarray studies, about 70-80% annotated genes are expressed under the same growth conditions as used in this study ([6] and our unpublished data). Nevertheless, our study suggests two ways to improve the results: the first is to more effectively remove rRNA or use a normalized cDNA library; the second is to employ "deep" sequencing techniques, either by performing multiple GS FLX runs, or by using Illumina [32] or ABI [33] methods which produce millions of reads, but of smaller average length.

## Conclusion

Our study indicated that there are still many genes missed in the initial genome annotation of *S. meliloti*. High-throughput sequence analysis of bacterial transcriptomes is feasible for the identification of new genes. Next-generation sequencing technologies will greatly facilitate the gene discovery process and improve genome annotation.

## Methods

### Cell culture and RNA isolation

*Sinorhizobium meliloti *strain1021 was grown at 30°C in TY medium [34] to mid-exponential phase (OD_600 _= 0.6). Cell growth was stopped by adding 1/9^th ^volume of stop solution (5% buffer equilibrated phenol pH 7.4 in ethanol) and placed on ice. Cells were collected by centrifugation in a microcentrifuge at maximum speed for 3 minutes. The cell pellets were stored in -80°C. Total RNA was isolated by using Qiagen RNeasy bacterial RNA purification kit (Qiagen, Valencia, CA). The total RNA was treated with DNase I on mini-RNeasy column before eluted with RNase free water. For RT-PCR experiments, an additional DNase I treatment was done after RNA was eluted from the RNeasy mini column to ensure that there was no genomic DNA contamination. 20 μl of total RNAs eluted from the RNeasy mini-column were treated with 5 μl DNase I (Qiagen) in 10 μl RDD buffer, 1 μl RNase inhibitor (Invitrogen, Carlsbad, CA) and 64 μl RNase free water (Qiagen) at 25°C for 30 minutes. The RNAs were then extracted with phenol/chloroform and precipitated with ethanol using standard protocols. 16s and 23s rRNAs were then depleted using the MICROBExpress™ Bacterial mRNA Enrichment Kit (Ambion, Austin, TX). Total RNAs and rRNA depleted RNAs were quantified and analyzed on the Agilent 2100 Bioanalyzer. 7 μg of total RNA per reaction was used. After 16s and 23s rRNAs were depleted, about 0.5 μg (7%) RNAs was recovered. The total RNA samples had RNA integrity number (RIN value) of 8.0 or better. As shown in Figure 2, more than 90% 16s and 23s rRNAs were depleted. We analyzed more than 10 independent rRNA-depleted preparations on the Agilent 2100 Bioanalyzer. The results were consistent and showed that the 16s and 23s rRNA peaks were greatly reduced in these preparations but not completely removed (Figure 2). In addition, two small peaks immediately before 16s and 23s rRNAs could not be removed by the Ambion MicrobExpress kit. The two peaks were consistently present in all of our RNA preparations.

Two RNA samples were prepared for RNA amplification. Sample 1 was 16s and 23s rRNA depleted RNA sample as described above. Sample 2 was the 16s and 23s rRNA depleted RNA sample ligated to a 3' RNA adptor (5'-PO_4_-UUCGCUGUUC UUAGCGGCCG CAUGCUC-idT-3'; idT: 3' inverted deoxythymidine) (Dharmacon Research, Lafayette, CO) and a 5' RNA adptor (5'-OH-AUGUGCGCGA CUUCCUGUAG ACGGAACGCU AGAAGAAA-OH-3') (Dharmacon Research). 3' and 5' adaptor ligations were done as described in Argaman *et al*. 2001 [35].

### RNA amplification and cDNA preparation

To obtain enough cDNA for sequencing, the 16s and 23s rRNA depleted RNAs (sample 1 and 2) were amplified using Nugen WT-Ovation Pico RNA amplification system [36]. 5 ng of starting RNA was used. The SPIA™ amplified single strand cDNA (2.5 ug) was then taken through a second strand cDNA synthesis, using the following conditions: 5X 2nd strand reaction mix 30 μl (Invitrogen), dNTP, 10 mM 3 μl (Invitrogen), *E. coli *DNA ligase 1 μl (Invitrogen), *E. coli *DNA polymerase I 4 μl (Invitrogen), RNase H 1 μl (Invitrogen), RNase-free water 91 μl (Ambion). The reaction mix was incubated at 16°C for 2 hours. The cDNA was then purified using the Qiagen PCR clean up kit resulting 4 ug of cDNA quantified by using the Nanodrop spectrophotometer. 1 ug of cDNA of each sample was size selected (>100 bp) using Roche's GS FLX library Preparation Guide recommendations (no nebulization was necessary due to the size range of the cDNA GS FLX library Preparation Guide), and a single stranded library was created.

### GS FLX sequencing and data filtering

The DNA sequencing libraries for the two samples were combined with the sequencing beads in 4 different concentrations to determine the optimal conditions for emPCR amplification. All 8 preparations were sequenced in 8 lanes of a GS FLX sequencing plate using the standard Roche/454 protocols. Sequencing data was obtained after a 7 hour run on the GS FLX. The 54,162 raw reads from GS FLX sequencing run that passed the sample key code filter (initial bases TCAG) were further filtered by the 454 software to eliminate 10,521 mixed reads (with two or more different DNA strands/bead), 15,604 excessively short reads (less than about 50 bp), and 9,032 interrupted reads ("dots"). 35% of the raw reads passed all filters in this titration run to provide the 19,005 high quality reads used in this study.

### Sequence analysis and mapping to *S. meliloti *genome

GS FLX sequences passed filtering criteria were BLASTN-aligned to *S. meliloti *genome. Sequences with matching to rRNA operons were filtered. The remaining sequences were BLASTed against RefSeq genes and our predicted new genes from our gene annotation pipeline.

### RT-PCR

5 μg DNase I treated total RNA was reverse transcribed using superscript II with 4 pmoles of equally mixed gene-specific primers for each candidate gene selected (cm012b-cm0031b, Table S1). Primers were designed using Primer3 [37]. For PCR, each 40 μl reaction includes 0.5 μl of 40 μl reverse transcription reaction, 20 μl of 2X GoTaq Green Master Mix (Promaga, Madison, WI) and 0.5 μM of primer pair of each gene (IDT, Coralville, IA). PCR conditions were 95°C 2 min, 30 cycles of 95°C 45 s, 52°C 45 s, 72°C 60 s, and a final cycle of 72°C for 10 min. PCR products were examined by electrophoresis in a 2.5% agarose/TAE/EtBr gel. Sequencing was performed using the BigDye Terminator Cycle Sequencing Kit (Applied Biosystems) and analyzed on an Applied Biosystems model 3730 automated capillary DNA sequencer.

## Authors' contributions

CM designed and executed the experiments, performed data analysis and drafted the manuscript. CE developed the RNA amplification method and performed RNA amplification and cDNA sample preparation for GS FLX sequencing. RVJ helped with data analysis. CE and RVJ wrote portions of the Methods. BWSS and RVJ critically edited and revised the manuscript. BWSS provided funding, coordination and oversight of the project. All authors read and approved the final manuscript.

## Supplementary Material

Additional file 1 Supplementary Table S1. New protein-coding genes predicted by PATRIC that are located in the intergenic regions Click here for file

Additional file 2 Supplementary Table S2. Primers Click here for file

Additional file 3 Supplementary Table S3. Genes with high copy number of GS FLX sequences Click here for file
